# Understanding the proliferation of bacteria across anode surfaces in microbial fuel cells (MFCs)

**DOI:** 10.1007/s00253-025-13653-5

**Published:** 2025-12-08

**Authors:** Hannah Bird, Ben Allen, Sharon B. Velasquez-Orta, Elizabeth Heidrich

**Affiliations:** School of Engineering, Newcastle University, Newcastle Upon Tyne, NE1 7RU UK

**Keywords:** Microbial fuel cells, Biofilm formation, Microbial community assembly, Electrogenic biofilms, Spatial and temporal dynamics

## Abstract

**Abstract:**

Microbial fuel cells (MFCs) offer a promising alternative for sustainable wastewater treatment and energy recovery. However, the mechanisms underpinning electrogenic biofilm formation remain poorly understood. This study investigates the spatial and temporal dynamics of microbial community assembly using a novel multi-electrode MFC design under two substrate conditions: acetate and starch. Pre-inoculation of three designated electrodes led to successful current generation within 110 h in both MFCs, while a dispersed inoculation strategy failed to establish electrogenic biofilms despite equivalent inoculum volume. Electrode positioning significantly influenced start-up, with vertical alignment above inoculated electrodes facilitating faster colonisation and current generation than lateral spacing. Notably, starch-fed MFCs exhibited more rapid and widespread biofilm proliferation, suggesting that complex microbial consortia may disperse more efficiently than single-function electrogens. Community sequencing revealed spatial heterogeneity and a shift from diverse to more optimised anodic communities over time. *Geobacter* initially dominated, but community succession was shaped by substrate complexity, competition, and spatial structure. Interestingly, non-inoculated electrodes often outperformed inoculated ones, indicating that deterministic selection pressures favoured more efficient biofilms. However, long-term current production declined, particularly under batch conditions, suggesting that population drift and limited microbial renewal limited sustained performance. This study is the first to characterise electrogenic biofilm assembly in a multi-electrode MFC, highlighting the interplay between stochastic dispersal and deterministic selection. These findings underscore the importance of inoculation strategy, substrate selection, and continuous microbial replenishment for optimising MFC performance and real-world applicability.

**Key points:**

• *Substrate complexity shaped colonisation and distinct microbial communities.*

• *Vertical electrode positioning enhanced colonisation and start-up efficiency.*

• *Temporal succession led to specialised but less diverse electrogenic biofilms.*

**Supplementary Information:**

The online version contains supplementary material available at 10.1007/s00253-025-13653-5.

## Introduction

The rising demand for clean energy has sparked interest in the use of alternative technologies. Greenhouse gas emissions from the water sector are already significant, at levels comparable to the aviation industry (Dunn et al. 2024). Conventional wastewater treatment methods such as activated sludge (AS), are energy-intensive, with aeration alone consuming ~ 50% of a plant’s total electricity use (Larzillière et al. 2024). Microbial fuel cells (MFCs) offer a promising dual-purpose solution: treating wastewater while generating renewable energy. However, the basic microbial processes governing their performance remains poorly understood. While both the anode and cathode electrodes are important (Zhou et al. 2011), the biological anode is arguably the most crucial part of the system. Here, the bacteria attach and grow and metabolise pollutants to generate a current (Yaqoob et al. 2020). Anodes have therefore attracted an ever-increasing interest due to their significance in extracellular electron transfer (EET) and pollutant removal. While recent modifications to anodes have enhanced MFC efficiency, lab-scale experiments continue to reveal considerable performance variations both between different anodes and across individual anode surfaces (Hindatu et al. 2017). These disparities are largely attributed to biological factors, primarily uneven bacterial colonisation, electron transfer inefficiencies, and biofilm formation variability across the anode surface (Santoro et al. 2017). Addressing these challenges is critical for achieving reliable and efficient power generation, as localised inefficiencies will become more pronounced when scaling up these systems.

A key factor influencing MFC performance is the development of a stable and electroactive biofilm on the anode. The formation and stability of these biofilms are influenced by four key ecological processes from Vellend’s framework: dispersal, speciation, selection, and drift (Vellend 2010) (Table 1). While biofilm formation is often discussed in the context of optimising electrogenic bacterial populations, both electrogenic and non-electrogenic bacteria colonise the anode, influencing biofilm structure and function. During colonisation, stochastic fluctuations (drift) and selective pressures interact to shape microbial community development, ultimately impacting system stability and performance (Yanuka-Golub et al. 2021; Zhou et al. 2013). Biofilms that form through more deterministic, selection-driven processes, such as microbial competition, often exhibit greater efficiency (Zhang et al. 2019). However, the ability to actively manage biofilm formation in MFCs remains a challenge, as the factors influencing early-stage microbial colonisation are not yet fully comprehended (Yanuka-Golub et al. 2021).

**Table 1:** Vellend’s processes for a robust mixed community assembly (Nemergut et al. 2013; Larzillière et al. 2024) and their possible significance in MFCs

Process	Description	Possible significance in MFCs
Dispersal	Movement of microbes across space, including the arrival of new microbial strains	Dispersal via incoming wastewater or bioaugmentation could enhance microbial diversity, supporting stable biofilms
Speciation	Genetic variation giving rise to new microbial populations	Introduction of gene diversity could lead to a more adaptable and productive microbial community over time
Selection	Changes in community structure driven by variations in fitness, causing certain species to be favoured	Optimising conditions such as pH and substrate type could enhance selection for EAB (e.g., *Geobacter*)
Drift	Stochastic (random) changes in the relative abundances of different microbial communities through time	Controlled drift could increase microbial diversity, promoting community stability

Understanding the roles and interactions of Vellend’s ecological processes in MFCs could provide valuable insights into how electroactive microbial communities can be engineered or assembled to enhance resilience and stability for wastewater treatment applications. One of the primary obstacles in MFC research is achieving consistent replication between reactors. Dispersal and stochasticity during colonisation contribute to inconsistent community development, even under identical conditions. Attempts to standardise colonisation (e.g., biofilm reseeding) have shown limited reproducibility, underlining the influence of spatial and compositional factors on microbial community assembly (Leicester et al. 2023).

This study investigates bacterial proliferation across anode surfaces in MFCs using a novel multi-electrode configuration. The research aims to provide novel insights into the temporal and spatial dynamics of biofilm formation and its impact on system performance. To achieve this, three MFCs, each with 25 anodes, were designed to assess the effects of inoculum distribution and substrate complexity. One reactor (MFC_Dispersed_) was uniformly inoculated with a return sludge liquor (RSL)-acetate mix; while the other two (MFC_Acetate_ and MFC_Starch_) received only partial inoculation on three anodes and were fed acetate and starch, respectively. To explore microbial colonisation dynamics and performance variability across anodes, the following hypotheses were tested:

1. Electrode activation in a dispersed inoculum system (MFC_Dispersed_):
  *H1*: Various anodes within the MFC_Dispersed_ system will initially generate current due to random interactions driven by stochastic EAB-electrode interactions.
  *H0*: Current generation will exhibit non-random spatial patterns, indicating deterministic colonisation.
2. Current generation in directly inoculated anodes (MFC_Acetate_ and MFC_Starch_):
  *H2*: RSL-inoculated anodes in both MFC_Acetate_ and MFC_Starch_ will produce higher initial currents, with current generation gradually spreading to all anodes as biofilms develop and EAB proliferate.
  *H0*: Start-up times across clean anodes follow an exponential distribution, consistent with a constant microbial assembly rate ($\lambda =1/\overline{t}$).
3. Electrogenic biofilm optimisation on distant anodes (MFC_Acetate_ and MFC_Starch_)
  *H3*: Over time, non-inoculated electrodes in MFC_Acetate_ and MFC_Starch_ will surpass the performance of inoculated ones, with this effect being more pronounced in MFC_Acetate_ due to acetate’s direct utilisation by EAB. Electrogenic biofilms on clean anodes set away from the inoculated anodes will be more optimised, driven by deterministic factors such as selective pressure and microbial competition, favouring electrogenic proliferation
  *H0*: No performance differences will emerge between anodes, suggesting limited selective optimisation.

## Material and methods

### Reactor configuration

**Fig. 1:**
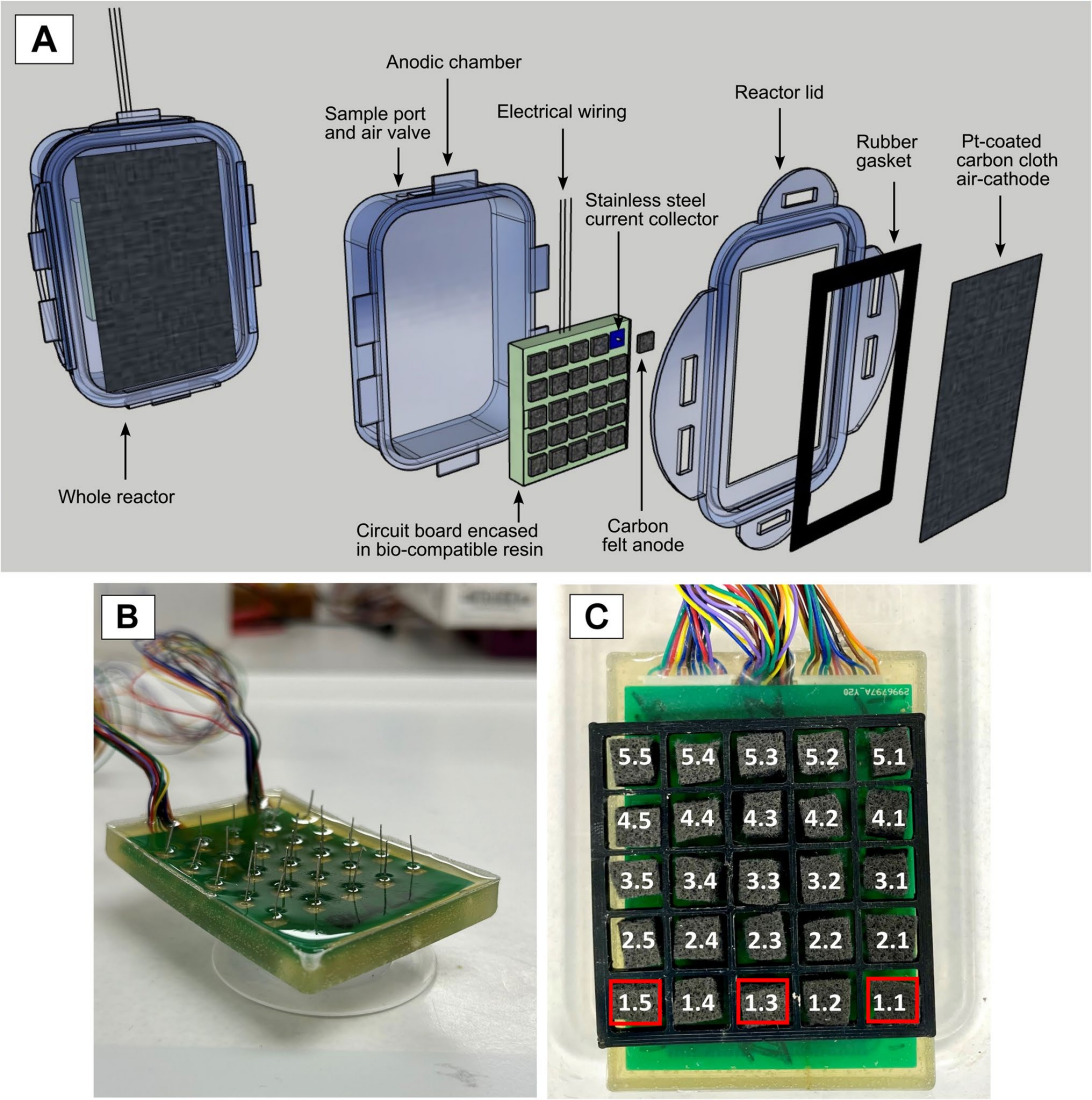
**A** 3D sketch of the single-chambered air–cathode MFC design (left) and in an exploded view to show the individual components (right). **B** Circuit board configuration with stainless steel ‘pin’ current collectors set in epoxy resin. **C** Anode ‘windowpane-like’ arrangement with a 3D-printed grid. Numbers indicate electrode labels, where the first digit represents the row and the second digit represents the column (e.g., Electrode 1.5 is in row 1, column 5). Red boxes represent inoculated electrodes

A novel multi-electrode MFC was designed and constructed for this study. Three reactors were constructed from inexpensive plastic food containers with an air–cathode configuration (Fig. 1). The plastic containers were chosen due to their easy-to-open and leakproof design (LocknLock Ltd, Korea). Each reactor consisted of a single anodic chamber with a total working volume of 310 mL. The air–cathode electrode was a 0.5 mg/cm^2^ 20% Platinum loaded Vulcan carbon cloth (Fuel Cell Store®, USA) and had a surface area of 40 cm^2^. The cathode cloth was glued onto a rubber gasket (platinum side facing the air), which was glued onto the lid of the plastic reactor using Gorilla Epoxy Resin (Gorilla Glue Ltd, UK) to provide a watertight seal.

The multi-electrode design consisted of a custom designed circuit board (Fig. 1B; details of which can be found in Supplementary Information, S.1), where 25 individual carbon felt anode squares (6 mm × 6 mm × 3 mm) were positioned in a windowpane-like arrangement across a single surface (SGL Carbon, Germany) (Fig. 1C). Pieces of stainless-steel wire (Clarke © Tools, Chronos Ltd, UK) measuring 2 mm in length were used as current collectors, connecting the anodes to the circuit board. These stainless-steel ‘pins’ were soldered onto the circuit board using a low temperature solder paste (Chip Quik, Canada). Electrical wire (30AGW) was also soldered to the circuit board to connect all individual anodes to the computer software. A silicone mould (Easy Composites Ltd, UK) was made and used to encase the wired-up circuit board in a translucent epoxy (M.G. Chemicals, Canada), protecting all electronic assemblies from any liquids. The individual anodes were then glued into place on each stainless-steel pin using a conductive epoxy glue (M.G. Chemicals, Canada). To ensure the individual anodes did not touch, a 3D-printed grid was made, separating them by 2 mm.

### Reactor inoculation and operation

Three multi-electrode MFCs were designed to assess how both inoculum density and substrate complexity influence biofilm development and current generation. The MFCs differed in their inoculation strategy and substrate type. In MFC_Dispersed_, 31 mL (10% by volume) of RSL (high-strength wastewater obtained from Howdon STW, Newcastle, UK) was mixed with acetate and fully dispersed across the reactor, ensuring uniform microbial exposure across all anodes. In MFC_Acetate_ and MFC_Starch_, only three carbon felt anodes at the reactor base were inoculated, while the remaining 22 anodes remained clean to allow monitoring of biofilm proliferation over time. For these reactors, the same inoculum volume used in MFC_Dispersed_ (31 mL) was centrifuged to form a pellet, re-suspended in a smaller liquid volume, and manually injected into three anodes with a syringe to ensure uniform distribution and absorption. The inoculated anodes were briefly dried at room temperature prior to reactor assembly, after which the reactors were filled with the designated feedstock solution (acetate for MFC_Acetate_, starch for MFC_Starch_). Both acetate and starch feedstocks were prepared at 500 mg/L COD, and consisted of: 0.528 g/L NaH_2_PO_4_∙H_2_O, 0.984 g/L Na_2_HPO_4_, and either 1 g/L NaC_2_H_3_O_2_ (acetate) or 0.25 g/L of starch. Additionally, 1.5 mL of both vitamin and trace element solution (ATCC, Teddington, UK) were added. All chemicals were of analytical grade (Sigma-Aldrich). Detailed inoculum composition and electrode-specific dosing are provided in the Supplementary Information (Table S1).

All reactors were sparged with ultra-high purity (UHP) nitrogen gas (99.998%) for 20 min to remove dissolved oxygen from the influent and establish anaerobic headspace conditions. Gas bags filled with nitrogen were attached to the reactors to maintain these headspace conditions during operation. Reactors were positioned vertically and operated in batch mode at 22 °C in an incubator. MFC_Dispersed_ ran for 38 days (4 feed cycles), while MFC_Acetate_ and MFC_Starch_ ran for 109 and 88 days, respectively (12 feed cycles each). Feed cycles were determined by the complete depletion of the substrate, which was determined from the temporal current profiles. The current output of each individual anode was continuously monitored using LabVIEW software, and data was processed in R Studio (version 4.4.1).

### Chemical measurements

To chemically characterise the anolyte, the following tests were conducted: chemical oxygen demand (COD), pH, conductivity, and dissolved oxygen (DO). These were measured at both the beginning and end of each test. COD was estimated using the HACH-Lange LCK 114 photometric test kits according to standard methods. A Jenway 3310 digital pH probe and HACH HQ30d conductivity and DO probes were used to measure these parameters.

### Microbial community analysis

**Fig. 2:**
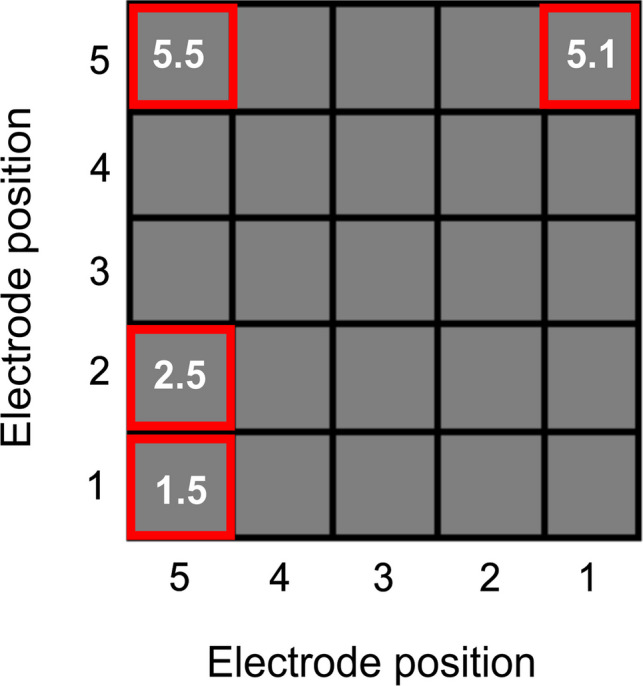
Position of electrodes sampled for DNA sequencing midway through the experiment in MFC_Acetate_ (Feed 6) and MFC_Starch_ (Feed 5)

The microbial communities of MFC_Acetate_ and MFC_Starch_ were analysed both midway through and at the end of the experiment. During this process, the systems were accessed via the clip-lid, and 2-mm sections of carbon felt were aseptically removed from the upper-left corner of the selected anodes in MFC_Acetate_ and MFC_Starch_ (Fig. 2). Midway sampling was designed to be rapid and minimally invasive to avoid disrupting biofilm development. To further reduce potential disturbance, only four anodes were sampled. These were selected based on their positional variation, enabling assessment of community spread within the reactor and evaluation of the experimental hypotheses:

- 1.5 – Inoculated electrode
- 2.5 – Electrode directly adjacent to the inoculated electrode
- 5.5 – Electrode positioned away from the inoculated electrode
- 5.1 – Electrode exhibiting low to no current in MFC_Acetate_ and poor performance in MFC_Starch_

These samples were collected at the start of Feed 6 (day 54) for MFC_Acetate_ and at the start of Feed 5 (day 32) for MFC_Starch_.

All biofilm samples (midway and end) were stored in 0.5 mL of DI water at − 20 °C. Samples of the RSL used for inoculation were also preserved for sequencing. Microbial community analysis was conducted by extracting DNA using the DNeasy PowerLyzer PowerSoil kit (Qiagen, UK) following the manufacturer’s protocol. Sequencing was performed at NU-OMICS (Northumbria University, UK), using the Earth Microbiome 16S amplicon protocol (v2), which targets the hypervariable V4 region of the 16S rRNA gene (Caporaso et al. 2011). Amplification was carried out using primers 515 F (GTGYCAGCMGCCGCGGTAA) (Parada et al. 2016) to 806R (GGACTACNVGGGTWTCTAAT) (Apprill et al. 2015). Library preparation and sequencing were conducted on the Illumina MiSeq platform using V2 500-cycle chemistry (Illumina, UK). Thermocycling conditions were as follows: initial denaturation at 94 °C for 3 min; 35 cycles of denaturation at 94 °C for 45 s, annealing at 50 °C for 60 s, and extension at 72 °C for 90 s; followed by a final extension at 72 °C for 10 min. Amplicon sequence variants (ASVs) were identified using the DADA2 pipeline (Callahan et al. 2016), and taxonomic classification to the genus level was performed using the SILVA rRNA gene database (v 132) (Yilmaz et al. 2014). Downstream analysis was carried out using the *phyloseq* package in R (McMurdie & Holmes 2013), and data were normalised to an equal sampling depth using the R function rarefy_ even_sampling_depth.

### Statistical analysis

To investigate the temporal dynamics of microbial community assembly, start-up times (time to > 0.03 A/m^2^) of non-inoculated anodes in MFC_Acetate_ and MFC_Starch_ were analysed. Assuming a constant microbial assembly rate, start-up times were modelled using an exponential distribution, with the rate parameter λ estimated as $\lambda =1/\overline{t}$, where $\overline{t}$ is the sample mean. Goodness-of-fit was assessed using Kolmogorov–Smirnov (KS) and chi-square tests, with expected frequencies derived from the estimated exponential distribution. Degrees of freedom for the chi-square test were defined as *k* – 2 to account for λ estimation and sample size.

Community differences were visualised via PCoA (Bray–Curtis dissimilarity), and tested using PERMANOVA (*adonis2*, R v4.4.1). Significance was based on 999 permutations (*p* < 0.05), with R^2^ indicating effect size. Analyses used *phyloseq*, *vegan*, and *ggplot2*.

## Results

This study uses a unique multi-electrode configuration, consisting of 25 individual anodes arranged in a ‘windowpane-like’ design across a single surface. The experiments aimed to investigate bacterial proliferation across anode surfaces in MFCs under varying substrate complexities, providing insights into the spatial and temporal dynamics of biofilm formation and its impact on system performance.

### Electrode start-up and current generation

#### Dispersed inoculum system

**Fig. 3:**
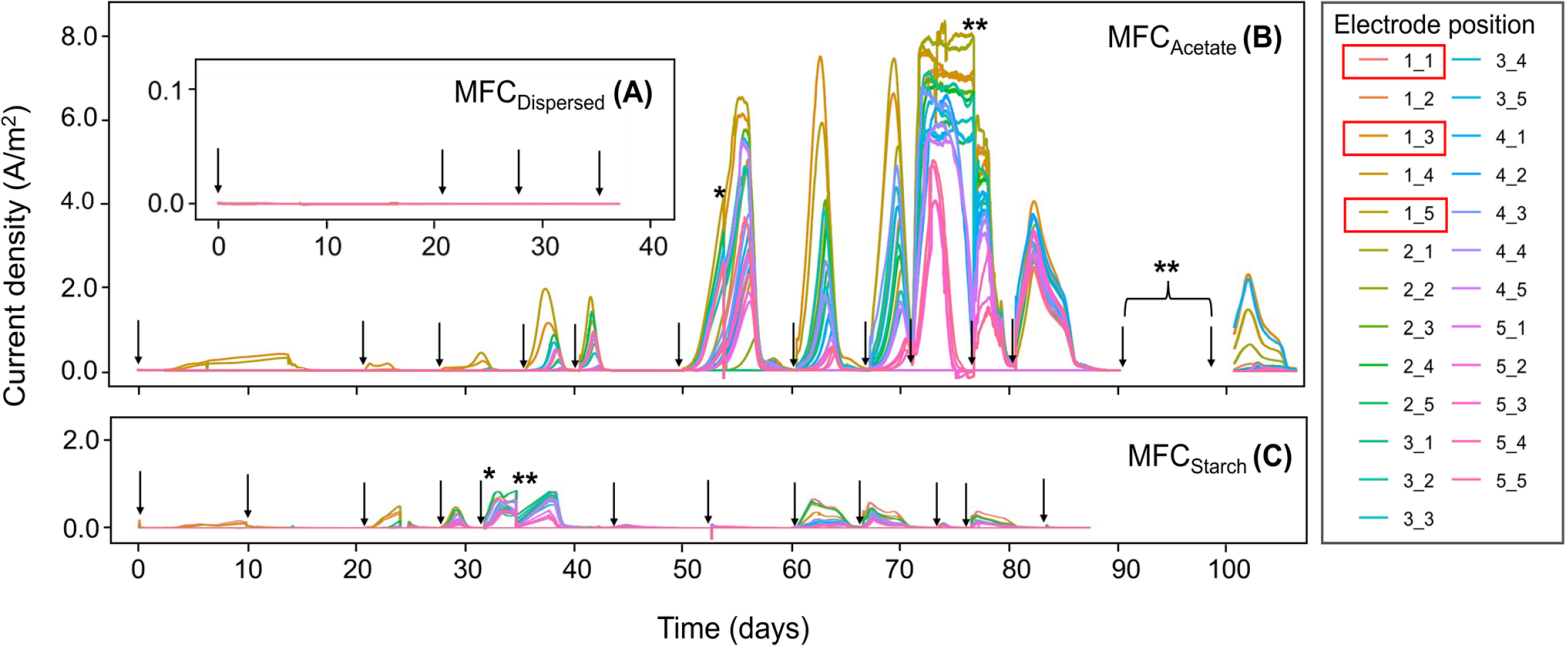
Current outputs for **A** MFC_Dispersed_ over 4 feeding cycles, **B** MFC_Acetate_ (3 RSL-inoculated electrodes) and **C** MFC_Starch_ (3 RSL-inoculated electrodes) both over 12 feeding cycles. Arrows indicate the addition of fresh anolyte (acetate or starch). Electrode positions are labelled based on row and column coordinates, where the first digit represents the row number, and the second digit represents the column number. Red boxes highlight inoculated electrodes in MFC_Acetate_ and MFC_Starch_. Asterisk (*) denotes biofilm extraction on selected anodes. Double asterisk (**) denotes software issue

In MFC_Dispersed_, a dispersed inoculum system, 31 mL of RSL (10% of the reactor volume) was introduced into an acetate feedstock to a total volume of 310 mL. This inoculum volume was constrained to match the total amount absorbed into the three pre-inoculated anodes in the directly inoculated systems. It was anticipated that this volume would be sufficient to produce current based on studies using raw wastewater, with the assumption that RSL would be more bacteria rich. Following inoculation, the reactor was operated under temperature-controlled conditions for 21 days. However, no current was detected across any of the 25 electrodes during this period (Fig. 3A). To assess whether bacterial attachment had occurred but there was simply not enough time or substrate availability to initiate growth and current, the MFC was emptied and re-fed with pure acetate over three additional feed cycles. Despite this, no current was generated throughout the 38-day operational period.

This MFC system was designed to test H1, which proposed that stochastic interactions between EAB and electrode surfaces would lead to variable current generation across anodes in a dispersed inoculum system. As monitoring the proliferation of bacteria across the anode surface was the main focus of this study, this reactor set-up was not pursued further.

#### Directly inoculated systems

The performance of the directly inoculated MFCs, in terms of current production was investigated over 12 feeding cycles (Fig. 3B and C).

**Fig. 4:**
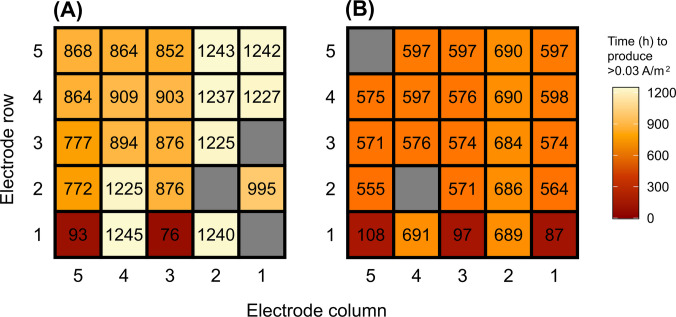
Heatmap showing time taken in hours for **A** MFC_Acetate_ and **B** MFC_Starch_ to produce > 0.03 A/m^2^ current. The grey squares denote electrodes in OCV due to no connection

**Fig. 5:**
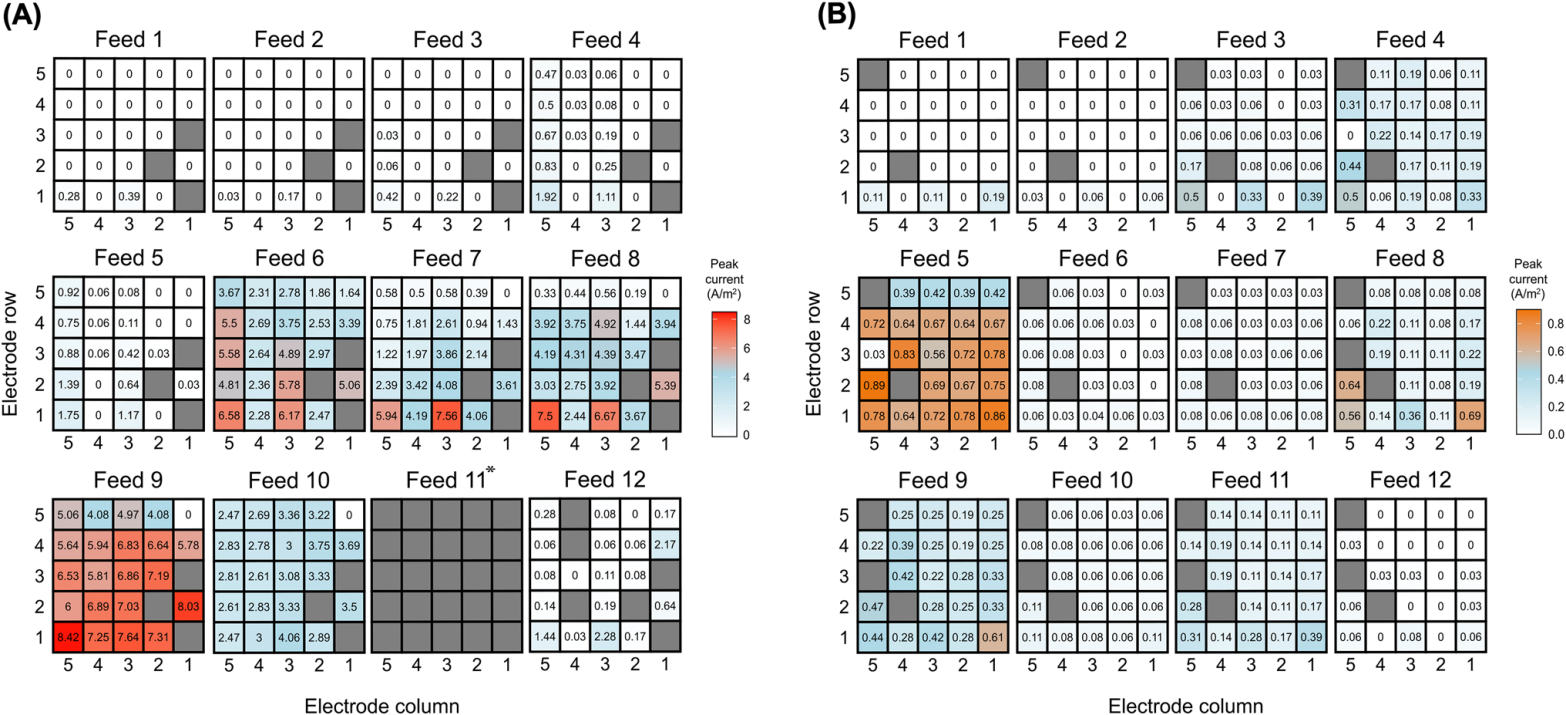
Heatmaps showing the peak current outputs of the individual electrodes across 12 feeding cycles for **A** MFC_Acetate_ and **B** MFC_Starch_. The grey squares denote electrodes in OCV. *Current outputs for Feed 11 in MFC_Acetate_ were not recorded due to a software issue. An animation of this time series can be found online at 10.25405/data.ncl.28592540

Current was first detected in MFC_Acetate_ after 3 days of operation (Fig. 3B). As expected under H2, the RSL-inoculated electrodes were the first to produce current, with electrodes 1.3A and 1.5A generating > 0.03 A/m^2^ after 76 and 93 h, respectively (Fig. 4A). These early signals of electroactivity support the prediction that directly inoculated anodes would exhibit higher initial currents. Due to a faulty connection, current outputs from electrode 1.1A (also RSL-inoculated) could not be recorded. Instead, this electrode remained in open circuit voltage (OCV) state, meaning it was electrically disconnected from the circuit and unable to contribute to current generation, effectively serving as a passive control. This was also the case for electrodes 2.2A and 3.1A. No current was detected from other electrodes during Feed 1, and this trend continued into the second feed cycle, further supporting the predicted difference between inoculated and non-inoculated electrodes. By Feed 3, two additional electrodes (2.5A and 3.5A), both positioned directly above electrode 1.5A, began to produce small amounts of current, consistent with gradual colonisation and current spread. This trend is visualised in the heatmap in Fig. 5A, highlighting the peak current outputs of the individual anodes for each feeding cycle.

After 37 days of operation (Feed 4), a sudden increase in the number of current-producing electrodes was observed, though the electrodes positioned laterally to the inoculated ones remained inactive (Fig. 5A). Instead, a clear pattern emerged where the electrodes positioned vertically above the inoculated ones, particularly in the fifth column (1–5.5A), generated higher currents, a trend that continued into Feed 5. To assess whether the start-up times of these current-producing electrodes followed a predictable pattern, statistical tests were conducted. The KS test and chi-square goodness-of-fit test for MFC_Acetate_ showed significant deviations from an expected exponential distribution (KS statistic: D = 0.532, *p*-value = 2.43 × 10⁻^5^ and chi-square statistic: X^2^ = 131.5, *p*-value = 2.75 × 10⁻^2^⁹), suggesting that electrode ‘start-up’ did not occur at a constant rate but rather followed a complex dynamic (see Supplementary Information, S.3).

A large increase in current outputs was observed across the electrodes in MFC_Acetate_ after 50 days of operation (Feed 6). All 22 functional electrodes were generating current with the inoculated anodes still producing the higher outputs, reaching peaks of 6.58 A/m^2^ (1.5A) and 6.17 A/m^2^ (1.3A). Similar to the previous two feeding cycles, the electrodes positioned vertically above the inoculated ones produced significantly higher currents than those positioned laterally (1.2A and 1.4A) (*p* = 0.0004, *t-*test), which were among the slowest to surpass outputs of > 0.03 A/m^2^. By Feed 7, a slight drop in peak current was observed across most anodes, with electrode 5.1A dropping from 1.64 A/m^2^ in Feed 6 to zero current production in Feed 7. Minimal changes occurred between Feeds 7 and 8, however, Feed 9 saw a substantial increase in current across all anodes, with electrode 1.5A achieving the highest peak current of all (8.33 A/m^2^). The heatmap for Feed 9 shows an almost even distribution of peak current outputs across all anodes, except for those positioned in the fifth row, which can be seen to produce slightly lower current outputs. The sudden drop in current on day 78 during Feed 9 was attributed to a software issue (Fig. 3B).

By Feed 10, current outputs had declined significantly, though the heatmap still indicated consistent performance across all electrodes. Due to a software issue, no data was recorded for Feed 11. Current generation further decreased during Feed 12, returning to levels seen in Feeds 4 and 5, with peak outputs of 2.3 A/m^2^ (1.3A) and 2.2 A/m^2^ (4.1A). By the end of Feed 12, three more electrodes lost connection, suggesting fouling of reactor components after 100 + days of operation.

In MFC_Starch_, current was first detected in the RSL-inoculated electrodes, 1.1S, 1.3S, and 1.5S, after 87, 97, and 108 h (3–4 days) of operation, respectively (Figs. 3C and 4B). This was similar to MFC_Acetate_. Faulty connections to electrodes 2.4S and 5.1S resulted in them operating in an OCV state. During the first feeding cycle, peak current outputs ranged from 0.11 to 0.19 A/m^2^ but reduced to 0.03–0.06 A/m^2^ during Feed 2 (Fig. 5B). A notable shift in reactor performance was observed during Feed 3, where all but four electrodes started generating > 0.03 A/m^2^ of current after 23 days. This was significantly faster than in MFC_Acetate_, where it took 37 days for the majority of electrodes to produce current (*p* < 0.0001, *t-*test). Like MFC_Acetate_, the electrodes in MFC_Starch_ positioned laterally to the inoculated ones (1.2S, 1.4S) were the slowest to surpass > 0.03 A/m^2^, taking 29 days. In contrast, those positioned vertically above not only had quicker start-up times (23 days, *p* < 0.0001, *t*-test), but also produced significantly higher current outputs (*p* = 0.02, *t*-test). This trend continued into Feed 4. Statistical analysis further supported these observations. The KS test and chi-square goodness-of-fit test showed significant deviations from the expected exponential distribution (KS statistic: D = 0.596, *p*-value = 1.36 × 10⁻⁶ and chi-square: X^2^ = 131.5, *p*-value = 5.28 × 10⁻⁶⁷), indicating that the start-up times of electrodes in MFC_Starch_ also did not follow a constant rate of microbial colonisation (see Supplementary Information, S.3).

By Feed 5, all functioning electrodes reached their peak current, doing so twice as fast as those in MFC_Acetate_. Most electrodes matched or exceeded the output of the inoculated anodes during this feed. However, peak currents were significantly lower (by an order of magnitude) compared to those in MFC_Acetate_ (*p* < 0.0001, *t-*test). Electrode 2.5S recorded the highest peak current during Feed 5 (0.89 A/m^2^), double that of the fifth-row electrodes (0.39–0.42 A/m^2^). The sudden drop in current observed in Fig. 4. 5 on day 34 was attributed to a software issue and lasted 4 days. A substantial drop in current outputs followed in Feeds 6 and 7, with several electrodes only reaching peak currents of 0.08 A/m^2^. Feed 8 saw a slight improvement, with two inoculated electrodes (1.1S and 1.5S) nearing their Feed 5 peak outputs (> 0.56 A/m^2^). However, the connection to electrode 3.5S was lost during this feed, preventing further current output from being recorded.

During Feed 9, the inoculated electrodes remained stable, while other electrodes showed an increase in current output. By day 74 (Feed 10), current dropped significantly across all electrodes (0.03–0.11 A/m^2^), though performance slightly improved in Feed 11. Similar to MFC_Acetate_, a sharp decline in performance was observed during the final feed (84 days of operation), with only nine electrodes remaining to produce current.

During the batch experiments, the average COD removals for MFC_Acetate_ and MFC_Starch_ were 77.1 ± 8.9% and 69.5 ± 8.7%, respectively (see Supplementary Information, Fig. S2).

### Spatial dynamics of microbial community assembly

**Fig. 6:**
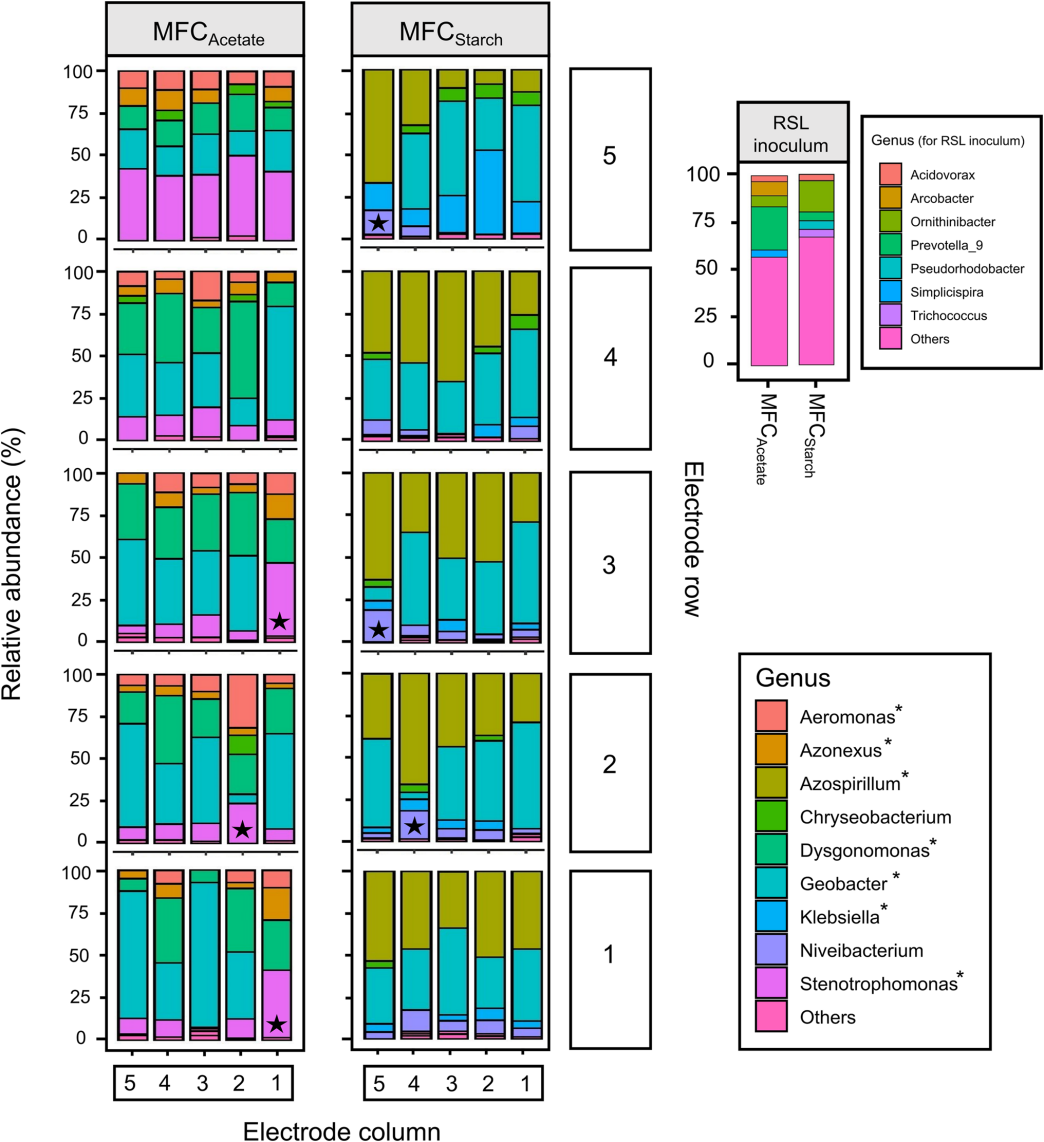
Bar charts showing the genus-level relative abundance of the 16S rRNA community profile within each anodic biofilm in MFC_Acetate_ and MFC_Starch_ at the end of Feed 12, along with the RSL inoculum communities. The bar chart layout corresponds to electrode positions. Genus with < 3% abundance are grouped under ‘Others’. Known EAB are marked with an asterisk (*), while electrodes in OCV conditions are indicated with a black star (★)

The microbial communities of the anodic biofilms in both MFC_Acetate_ and MFC_Starch_ were analysed using 16S rRNA gene sequencing. Figure 6 presents the microbial community structure of the electrodes after the final feed (Feed 12) at the genus level for both MFCs. As expected the microbial composition of the anodic MFCs differed from that of the original RSL inoculum. However, the two inocula used were relatively similar to each other, with an initial alpha diversity (Shannon index) of 4.26 ± 0.08. By the end of the experiment, the alpha diversity had decreased to 2.84 ± 0.33 in MFC_Acetate_ and 2.62 ± 0.30 in MFC_Starch_. *Geobacter* became the dominant genus in the anodes of both MFCs, with average relative abundances of 40.1 ± 20.1% in MFC_Acetate_ and 41.7 ± 15.5% in MFC_Starch_. In addition, *Dysgonomonas* (26.5 ± 11.8%) was the second most abundant genus in MFC_Acetate_, while *Azospirillum* (40.9 ± 16.7%) was the second most abundant in MFC_Starch_.

*Geobacter* was most abundant in the inoculated electrodes of MFC_Acetate_ which were fed acetate as the sole substrate, accounting for 85.4% of the community in electrode 1.3A and 74.9% in 1.5A, compared to lower levels in electrodes 1.2A (39.9%) and 1.4A (33.4%). A clear decline in *Geobacter* abundance was observed moving up the multi-electrode surface in MFC_Acetate_, decreasing from 58.3 ± 25.8% in the anodes of Row 1 to just 20.7 ± 4.4% in Row 5. Interestingly, *Geobacter* was significantly more abundant on electrodes positioned vertically above inoculated anodes compared to those positioned above non-inoculated ones (*p* = 0.028, *t*-test). In addition to *Geobacter*, the MFC_Acetate_ anodes were populated by other known EAB such as *Aeromonas* (9.8 ± 5.7%), *Azonexus* (7.3 ± 3.9%), and *Stenotrophomonas* (19.6 ± 14.8%). Notably, *Stenotrophomonas* dominated the anodes in Row 5, where its average abundance (41.2 ± 4.1%) was double that of *Geobacter*.

In MFC_Acetate_, three electrodes (1.1A, 2.2A, and 3.1A) operated in open OCV state which acted as controls, exhibited notable differences in microbial community composition compared to the other electrodes. These OCV electrodes showed less than 5% *Geobacter* coverage and an increased presence of other genera, such as *Stenotrophomonas*.

In MFC_Starch_, different trends emerged. The genus *Azospirillum* dominated in two of the inoculated electrodes, accounting for 45.2% and 53.5% of the microbial communities in 1.1S and 1.5S, respectively, while *Geobacter* predominated in 1.3S, making up 51.8% of the community (18% higher than *Azospirillum*). Unlike in MFC_Acetate_, *Geobacter* abundance increased progressively from the lower to the upper electrodes of the multi-electrode surface in MFC_Starch_, rising from 39.1 ± 8.5% in Row 1 to 47.1 ± 12.2% in Row 5. Additionally, no significant differences in *Geobacter* abundances were observed in MFC_Starch_ between electrodes positioned vertically above inoculated anodes and those above non-inoculated ones. The genus *Azospirillum* had consistent abundances across anodes in Rows 1–4, comprising of ~ 45% of the microbial communities and was most abundant in the OCV electrodes. However, its abundance dropped to 25% in Row 5, where *Klebsiella*, a known EAB, became five times more abundant compared to the other rows. The increased presence of *Klebsiella* on a distal, non-inoculated anode aligns with H3, which predicts that electrogenic biofilms may become more optimised on clean, distant electrodes over time due to selective pressures favouring optimised electroactive communities.

Similar to MFC_Acetate_, several electrodes in MFC_Starch_ were in OCV mode throughout the experiment (2.4S and 5.5S). While electrode 3.5S lost its connection after 60 days and subsequently entered OCV mode. Like in MFC_Acetate_, these electrodes showed a substantial reduction in *Geobacter* abundance (< 5%) and a rise in other genera, including *Niveibacterium* and *Azospirillum.*

### Temporal dynamics of microbial community assembly

**Fig. 7:**
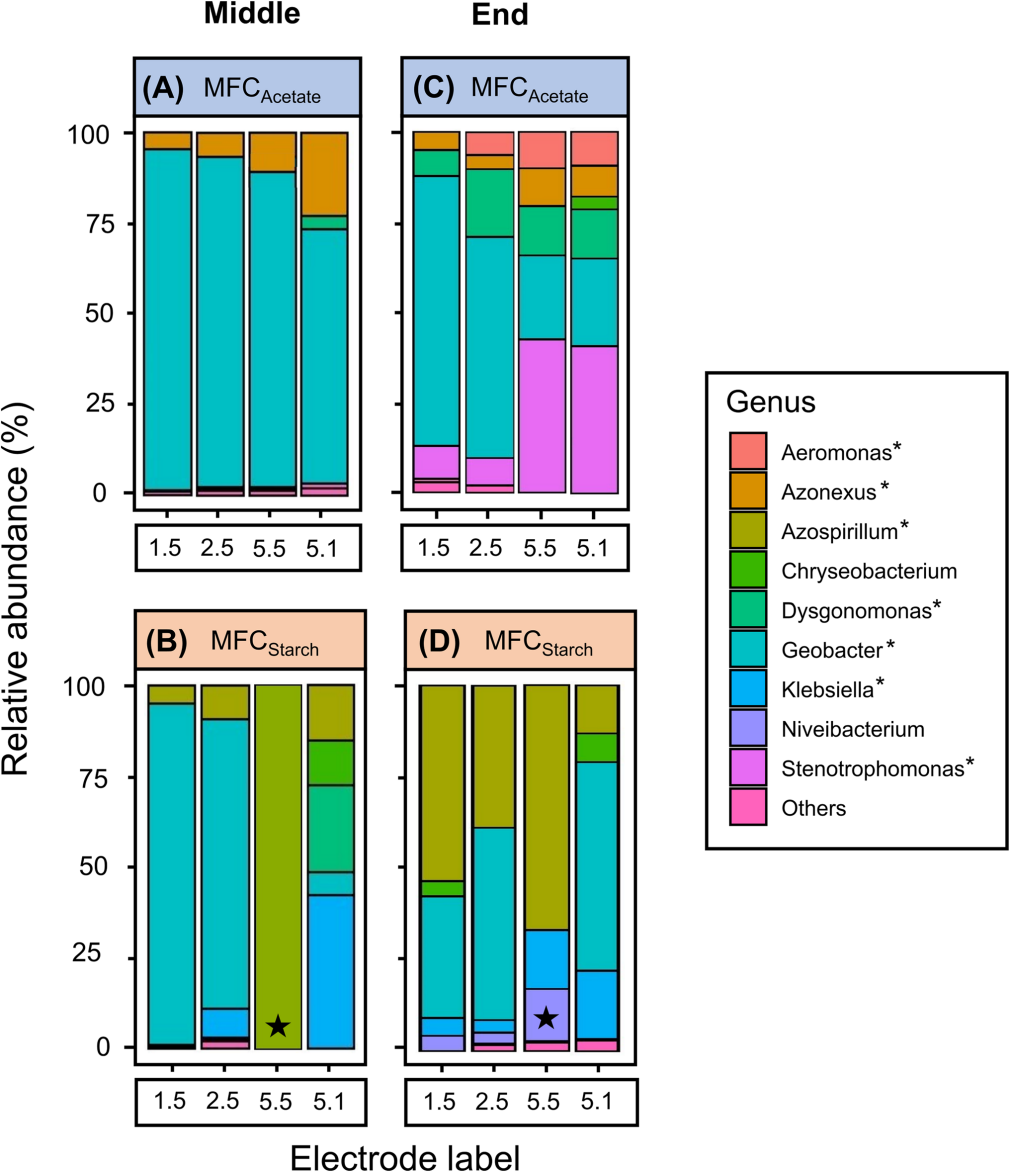
Genus-level relative abundance plots of 16S rRNA community profile for four selected anodic biofilms. Midway through the experiment, samples were taken at **A** Feed 6 for MFC_Acetate_ and **B** Feed 5 for MFC_Starch_. Corresponding anodic communities at the end of the experiment (Feed 12) are shown for **C** MFC_Acetate_, and **D** MFC_Starch_. Selection criteria for each anode are detailed in Fig. 2. Genera with < 3% abundance are grouped under ‘Others’. Known EAB are marked with an asterisk (*), while electrodes in OCV conditions are indicated with a black star (★)

Microbial communities from electrodes 1.5, 2.5, 5.5 and 5.1 were sampled from both MFC_Acetate_ (day 54, Feed 6) and MFC_Starch_ (day 32, Feed 5) after stable current generation. Community composition is shown in Fig. 7.

In MFC_Acetate_ during Feed 6, *Geobacter* dominated all sampled electrodes, reaching 96% abundance on the inoculated electrode 1.5A, which also produced the highest peak current. The abundance of *Geobacter* decreased with increasing distance from the inoculated electrode, dropping to 90% on electrode 2.5A, 86% on 5.5A, and 70% on 5.1A. This decline in *Geobacter* abundance correlated with reduced current production on the respective electrodes during this Feed. Small abundances of *Azonexus* were also detected in the electrodes at this stage. By the end of Feed 12 (54 days later), *Geobacter* abundance had declined across all four electrodes, dropping to 74.9% in 1.5A, 61% in 2.5A, and just over 20% in both 5.5A and 5.1A. This shift coincided with increased colonisation by other genera, such as *Aeromonas*, *Dysgonomonas*, and *Stenotrophomonas*, and a corresponding decline in current output.

Similar trends were observed in MFC_Starch_, where the inoculated electrode 1.5S exhibited 94% *Geobacter* coverage during Feed 5, which dropped to 33.3% by the final feed. In contrast, *Azospirillum* abundance increased by 48% over the same period. Interestingly, electrode 2.5S, despite having slightly lower *Geobacter* abundance in Feed 5 (79.6%), produced the highest peak current during that cycle. Electrode 5.5S, operated in OCV mode, contained only *Azospirillum* during Feed 5, but by Feed 12, small populations of other genera, including *Klebsiella* and *Niveibacterium*, had colonised the anode. Meanwhile, electrode 5.1S, a low-performing electrode, showed minimal *Geobacter* presence (6.1%) during Feed 5, yet this increased to 57.1% by Feed 12, despite the electrode failing to generate current.

These shifts in community structure were reflected in alpha diversity trends. In MFC_Acetate_, samples from Feed 6 exhibited intermediate diversity compared to both the initial inoculum and the final electrode communities, with a Shannon index of 2.02 ± 1.01. In contrast, MFC_Starch_ showed a different pattern, where alpha diversity levels at Feed 5 were actually higher than in the final electrode communities, with an average Shannon index of 2.94 ± 0.54.

**Fig. 8:**
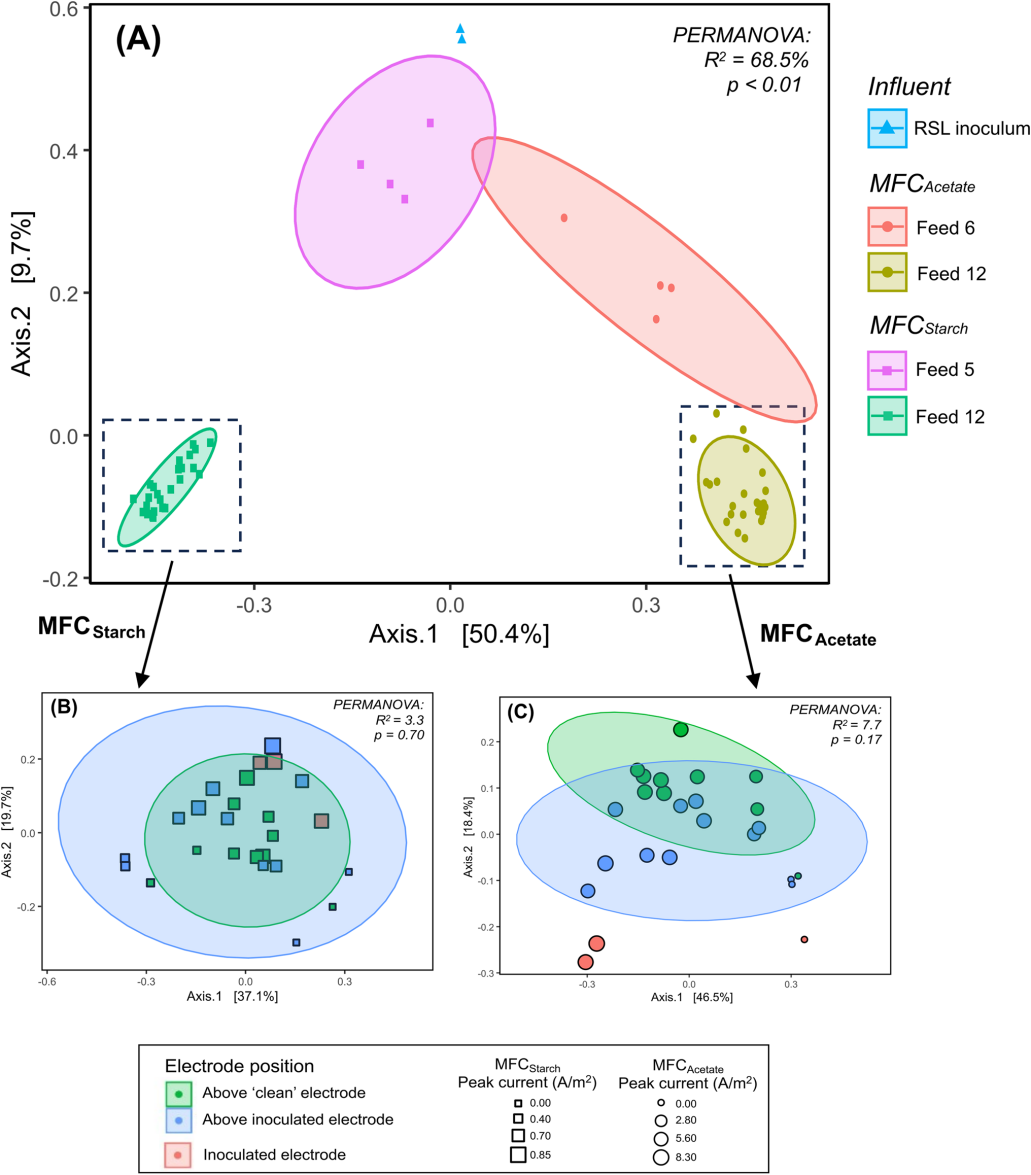
Principal Coordinates Analysis (PCoA) plots based on Bray–Curtis dissimilarity metrics, illustrating microbial community relationships. **A** Overall ordination of all microbial communities, including the influent. Microbial community composition at the end of the experiment (Feed 12) in **B** MFC_Starch_ and **C** MFC_Acetate_, grouped by electrode positioning above either inoculated or non-inoculated (clean) electrodes. Panels B and C are independent ordinations, not magnified views of plot A, and display new axis variations. Marker size represents peak current production, with larger markers indicating higher current output. Statistical significance of community differences was assessed using PERMANOVA

Multivariate ordination was conducted to evaluate differences in microbial community composition across samples. PCoA using Bray–Curtis dissimilarities revealed distinct clustering patterns, with increasing divergence between the influent RSL microbial communities and the electrode biofilms over time (Fig. 8A). Early biofilm samples (Feed 6, MFC_Acetate_; Feed 5, MFC_Starch_) clustered more closely with the influent communities, while samples collected after Feed 12 showed greater divergence, including clear distinctions between substrate types (acetate vs. starch). PERMANOVA analysis confirmed that 68.5% of the variance in microbial composition could be explained by both substrate and sampling time. The *p*-value (< 0.01) provides strong evidence that at least one of these factors significantly influenced community differences.

To further investigate microbial community variations, additional ordination was performed on the Feed 12 electrode samples from MFC_Acetate_ and MFC_Starch_, focusing on electrode positioning (Fig. 8B and C). PCoA showed no clear separation between microbial communities on electrodes positioned above inoculated versus above non-inoculated electrodes, as indicated by the overlapping ellipsoids. PERMANOVA analysis supported this, revealing no statistically significant differences (*p* > 0.05), with electrode positioning explaining only 7.7% of the variance in MFC_Acetate_ and 3.3% in MFC_Starch_. Additional factors, including electrode row position, were also tested but did not show significant effects on microbial community composition (Supplementary Information, Fig. S3).

## Discussion

This study provides novel insights into the temporal and spatial dynamics of electrogenic biofilm formation and its impact on reactor performance in a multi-electrode MFC. By examining biofilm start-up time, current generation, and microbial community assembly across MFCs using two substrates of varying complexity, this work evaluates the key processes influencing bacterial proliferation on anode surfaces.

In the directly inoculated systems (MFC_Acetate_ and MFC_Starch_), electrogenic biofilms established within approximately 110 h, as reflected in the initial current generation which was confined to the inoculated electrodes. This observation supports the hypothesis that pre-inoculating electrodes accelerates start-up time (H2). As the biofilms matured, current production progressively expanded across the multi-electrode surface. Notably, in both MFC_Acetate_ and MFC_Starch_, electrodes positioned vertically above the inoculated anodes exhibited significantly shorter start-up times and higher current outputs compared to laterally positioned electrodes. Statistical analysis using KS and chi-square tests confirmed that electrode activation did not occur at a uniform rate over time, rejecting the null hypothesis of random or exponential colonisation for both MFCs. Instead, start-up times followed more complex spatial and temporal patterns, suggesting that factors beyond simple diffusion-based dispersal influenced biofilm formation and current generation. Vertical positioning, rather than physical proximity alone, therefore, appears to play a key role in colonisation efficiency. One possible explanation could be in the slow and controlled refeeding strategy, which may have inadvertently promoted upward microbial movement along the electrode surface, thereby accelerating colonisation of electrodes located directly above the inoculated ones. Importantly, the inoculated electrodes were deliberately placed in the bottom row to eliminate the possibility of biofilm formation being driven by downward microbial settling. However, this upward tracking effect was not initially anticipated and warrants further investigation.

The start-up times for MFC_Acetate_ and MFC_Starch_ were similar at the inoculated anodes, though MFC_Acetate_ initiated start-up slightly earlier (by approximately 10–30 h). This was expected as acetate undergoes a single metabolic step directly carried out by electrogens, whereas starch requires sequential metabolic steps involving different microbial species (Velasquez-Orta et al. 2011). Despite this initial delay, electroactive behaviour proliferated more rapidly across the electrode array in the starch-fed MFC, with non-inoculated electrodes in MFC_Starch_ reaching the current performance benchmark (> 0.03 A/m^2^) approximately twice as quickly as those in MFC_Acetate_. This suggests that the dispersal of a metabolically diverse community capable of digesting a complex substrate occurs more readily than that of a single metabolic group. This result aligns to a degree with a previous study, where air–cathode MFCs fed with acetate took approximately twice as long to acclimate as those fed with starch (Bird et al. 2025). The delay was attributed to the initial scarcity of acetate-consuming electrogens and their lag phase before exponential growth, a pattern that also aligns with the current profiles observed in this study.

Overtime, non-inoculated electrodes not only matched but in some cases surpassed the current output of inoculated ones. This effect was most pronounced in MFC_Starch_, where the presence of a microbial community with greater diversity supported faster proliferation and eventual optimisation of electrogenic activity. In contrast, optimisation in MFC_Acetate_ was delayed, reflecting the dependence of acetate-fed systems on a narrower group of electrogens. These results indicate that deterministic processes, including substrate availability and microbial competition, were key drivers of biofilm assembly and optimisation, while stochastic dispersal alone was insufficient. Collectively, these findings provide partial support for H3, which proposed that electrogenic biofilms on non-inoculated anodes may, over time, outperform those on pre-inoculated ones.

Anodic biofilm composition revealed clear spatial heterogeneity across the electrode surfaces. In MFC_Acetate_, *Geobacter* abundance decreased with vertical distance from the inoculated row, whereas the opposite trend was observed in MFC_Starch_. Notably, in MFC_Acetate_, *Geobacter* was significantly more abundant on electrodes positioned directly above inoculated anodes than those above non-inoculated ones, although this effect was not statistically significant in MFC_Starch_. Despite these spatial variations, PCoA analysis revealed no clear separation between microbial communities on electrodes positioned above inoculated versus non-inoculated anodes. Vertical positioning explained only 7.7% of the variance in microbial community composition in MFC_Acetate_ and 3.3% in MFC_Starch_, while row positioning had no significant effect. These findings suggest that while spatial differences in *Geobacter* distribution were evident, broader microbial shifts were likely driven by a combination of other factors such as substrate complexity, selective pressures, and stochastic interactions (drift) rather than by electrode positioning alone.

Interestingly, electrodes in the uppermost row (Row 5) were populated by other EAB. In MFC_Acetate,_ *Stenotrophomonas* dominated (Shen et al. 2017; Venkidusamy & Megharaj 2016), whereas in MFC_Starch_, smaller abundances of *Klebsiella* were detected. As *Klebsiella* can directly utilise starch for electricity generation (Zhang et al. 2008), its presence on distant electrodes suggests that biofilm optimisation on previously uncolonised surfaces was not a random process. Instead, it was shaped by deterministic factors such as selective pressure and microbial competition, ultimately favouring electrogenic proliferation over time, again supporting H3. Control electrodes under OCV conditions exhibited noticeably low *Geobacter* abundances (< 5%) and an increased presence of other genera e.g., *Azospirillum* in MFC_Starch_, indicating a shift toward a passive microbial community in the absence of an applied voltage.

Microbial community analysis revealed significant differences between the inoculum, middle and final anode communities, highlighting the dynamic nature of biofilm succession. Alpha diversity analysis further revealed a decline from the influent to the final anode communities, indicating a shift towards a less diverse but more specialised microbial community. As the experiment progressed, microbial communities became increasingly distinct. In both MFCs, *Geobacter* abundance declined between Feeds 5–6 and Feed 12, coinciding with decreased power generation. This suggests increased competition between electrogenic and non-electrogenic bacteria, potentially reducing electron transfer efficiency. By Feed 12, *Geobacter* abundances were comparable in both MFC_Acetate_ and MFC_Starch_; however, the expected dominance of *Geobacter* across the whole acetate-fed system was not observed. PCoA clustering further revealed clear differences in community composition based on both substrate type (acetate or starch) and sampling time (inoculum, mid-point, and end), confirming that substrate availability and microbial competition were key selection pressures driving succession.

Together, these temporal and spatial shifts reinforce the hypothesis that biofilm development was shaped by a combination of stochastic dispersal events and deterministic selection pressures, where environmental factors and microbial interactions ultimately influenced species proliferation across the multi-electrode surfaces. To further evaluate the influence of stochastic processes in isolation, the MFC_Dispersed_ system was included as a contrasting condition. Results from this system revealed that electrode start-up did not occur as anticipated, contradicting the hypothesis that random interactions between EAB and electrode surfaces would be sufficient to promote biofilm formation.

Despite using the same RSL inoculum volume as in MFC_Acetate_ and MFC_Starch_, EAB attachment in MFC_Dispersed_ did not occur in any of the electrodes. This likely reflects dilution of the localised cell-surface interactions necessary for successful biofilm establishment and efficient electron transfer, suggesting that a minimum threshold concentration of EAB is required for anode activation. EAB are generally present in low abundance within natural inocula, e.g., Heidrich et al., (2016) estimated the most probable number (MPN) of effective electrogens in raw wastewater to be only 17 cells/mL, while other studies have reported higher values ranging from 1.0 × 10^3^ to 7.5 × 10^5^ cells/mL (Yang et al. 2016). However, these latter estimates are based on measurements of all electrogen content, not those able to initiate current. Based on the 31 mL of RSL used for inoculation (10% of the reactor volume), the effective electrogen count in MFC_Dispersed_ would equate to approximately 500 effective electrogens dispersed throughout the 310 mL MFC. It should be noted, however, that RSL is considerably more complex than raw wastewater, and thus the MPN derived from raw wastewater may not accurately represent its electrogenic potential.

Since dispersed inoculation is a common method in MFC studies, biofilm attachment would typically be expected; however, in this case, it did not occur. The strength of the inoculum, particularly its carbon content, plays a crucial role in biofilm development. Aiyer & Vijayakumar (2019) found that biofilm formation was strongest when undiluted wastewater was used as the inoculum, whereas increasing dilution resulted in poorly adhered biofilms. This suggests that dilution reduces the availability of essential microbial and organic components required for effective colonisation. In the case of MFC_Dispersed_, the combination of low EAB density and spatially dispersed microbial attachment likely hindered the initiation of electroactive biofilms, emphasising the importance of inoculum concentration and spatial distribution in MFC start-up dynamics. Additionally, electrode potential may also play a critical role in the formation of an electroactive biofilm, as studies have shown that applying a defined potential can stimulate biofilm development (Pinto et al. 2018), and this should be explored further in this system.

Overall, the observed microbial dynamics in this study can be attributed to five key factors, as summarised in Table 2. These findings highlight the complexity of microbial community assembly, revealing how both spatial positioning, substrate availability and temporal shifts interact to shape electrogenic biofilm development.

**Table 2:** Summary of the key factors influencing microbial community dynamics in the multi-electrode MFCs

Factor	Influence on microbial dynamics
Inoculation strategy	Pre-inoculating electrodes accelerated start-up, while dispersed inoculation likely diluted electrogenic interactions, delaying biofilm formation
Substrate complexity	The starch-fed MFC unexpectedly exhibited faster acclimation across the whole electrode surface Both acetate and starch-fed MFCs developed distinct microbial communities, indicating substrate-driven selection processes
Spatial positioning	Vertical positioning above inoculated electrodes, (rather than lateral proximity) enhanced colonisation and start-up efficiency, though this had minimal influence on the observed microbial variances
Selection pressures	Early *Geobacter* dominance was followed by shifts in microbial composition, suggesting biofilm maturation was shaped by competition and environmental adaptation
Temporal succession	Microbial communities diverged over time, with stochastic and deterministic processes such as microbial competition likely influencing biofilm development and electrogenic performance

Maintaining stable biofilm activity over extended operational periods proved challenging in this study. While both MFCs were consistently fed with fresh media over the 12 cycles, no additional inoculum was introduced. Initially, current production peaked, but it subsequently declined, and in the starch-fed system, it eventually ceased. This decline may be due to selection and drift within the existing population being insufficient to sustain an electroactive community under static conditions. These findings have significant implications for the long-term operation of METs, indicating that continuous or periodic replenishment of inoculum may be necessary to maintain biofilm function. This limitation is particularly relevant to laboratory studies operating under batch conditions, which fail to capture the dynamic microbial renewal observed in real-world systems.

This study is the first to utilise a multi-electrode MFC configuration to investigate the dynamics of microbial community assembly, providing novel insights into the spatial and temporal factors driving biofilm formation and electroactivity. The findings highlight the influence of inoculation strategy, substrate availability, spatial positioning and selection pressures in shaping biofilm development. Ultimately, biofilm maturation appears to be driven by a complex interplay between deterministic selection pressures (e.g., microbial competition and environmental factors such as substrate complexity) and stochastic dispersal events, highlighting the importance of both initial inoculation conditions and subsequent microbial interactions in electrogenic community assembly.

## Supplementary Information

Below is the link to the electronic supplementary material.

ESM 1 (DOCX 595 KB)

## Data Availability

The sequencing data that support the findings of this study have been deposited in the European Nucleotide Archive (ENA) under the accession number PRJEB95057.
