# Supplementary material for: Understanding the proliferation of bacteria across anode surfaces in microbial fuel cells (MFCs)
Source: Appl Microbiol Biotechnol. 2025 Dec 8;109:257. doi: 10.1007/s00253-025-13653-5 (PMC12689808; doi:10.1007/s00253-025-13653-5)
Supplement: Supplementary file 1 [file 253_2025_13653_MOESM1_ESM.docx]

**Supplementary Information**

**Understanding the proliferation of bacteria across anode surfaces in microbial fuel cells (MFCs)**

Hannah Bird^1^, Ben Allen^1^, Sharon Velasquez-Orta^1^, Elizabeth Heidrich^1*^

^1^School of Engineering, Newcastle University, Newcastle upon Tyne, NE1 7RU, UK

***Correspondence:**

Elizabeth Heidrich

elizabeth.heidrich@newcastle.ac.uk

# S.1 Multi-electrode circuit board details

A dedicated GitHub repository has been created containing all relevant information for the assembly of the multi-electrode circuit board:

<https://github.com/beadyallen/Muli-Electrode-MFC>

# S.2 Inoculum composition and electrode specific dosing

**Table S1** Inoculation strategy and substrate composition of the multi-electrode MFCs.

| **Reactor** | **Inoculum and substrate type** |
| --- | --- |
| MFC_Dispersed_ | *RSL-acetate mix (COD = 750 mg/L):*   - 279 mL acetate (90%) - 31 mL RSL (10% of reactor volume) |
| MFC_Acetate_ | *Individual electrode inoculation*  310 mL acetate medium (COD = 500 mg/L)  Solid fraction of RSL (31 mL centrifuged at 500 g for 5 mins) injected into three individual anodes (wet weight):   - Electrode 1.1A = 0.237 g of RSL - Electrode 1.3A = 0.290 g of RSL - Electrode 1.5A = 0.311 g of RSL |
| MFC_Starch_ | *Individual electrode inoculation*  310 mL starch medium (COD = 500 mg/L)  Solid fraction of RSL (31 mL centrifuged at 500 g for 5 mins) injected into three individual anodes (wet weight):   - Electrode 1.1S = 0.283 g of RSL - Electrode 1.3S = 0.384 g of RSL - Electrode 1.5S = 0.345 g of RSL |

# S.3 Statistical analysis – start-up time distribution

Analysis procedure:

- Collect start-up times in hours ($\overline{t}$).
- Estimate the rate parameter (λ) using the sample mean following the relationship $\lambda=1/\overline{t}$
- Perform goodness-of-fit tests (KS test and chi-square test).
- Evaluate *p-*values to assess the null hypothesis.

**MFC_Acetate_**

$\overline{t}$ = (1240, 1245, 995, 876, 1225, 772, 1225, 876, 894, 777, 1227, 1237, 908, 909, 864, 1242, 1243, 852, 864, 868)

- Mean start up time ($\bar{t}$) = 1016.95 h
- Estimated rate parameter (λ) = 0.000983 h
- **KS Test:**
  - *D* = 0.532
  - *p-value* = 2.43×10^-5^
  - The null hypothesis was rejected, indicating significant deviation between the empirical and theoretical CDFs
- **Chi-Square Test:**
  - X^2^ = 131.5
  - Degrees of freedom = 2
  - *p-value* = 2.75×10^-2^

Observed frequencies substantially deviated from the exponential model

**MFC3_Starch_**

- $\overline{t}$ = (689, 691, 564, 686, 571, 555, 574, 684, 574, 576, 571, 598, 690, 576, 597, 575, 597, 690, 597, 597)
- Mean start up time ($\bar{t}$) = 612.6 h
- Estimated rate parameter (λ) = 0.0016 h
- **KS Test:**
  - *D* = 0.596
  - *p-value* = 1.36×10^-6^
  - The null hypothesis was rejected.
- **Chi-Square Test:**
  - X^2^ = 305.2
  - Degrees of freedom = 2
  - *p-value* = 5.28×10^-67^
  - Strong evidence against the null hypothesis was observed

**Fig. S1** Goodness-of-fit tests for MFC_Acetate_ and for MFC_Starch_: Kolmogorov-Smirnov test comparing the empirical and theoretical cumulative distribution functions (CDF) for **a** MFC_Acetate_ and **c** MFC_Starch_, showing the degree of deviation between the observed and expected distributions. Chi-square goodness-of-fit test for **b** MFC_Acetate_ and **d** MFC_Starch_, illustrating the comparison between observed and expected frequencies across bins, with corresponding p-values indicating the fit to the exponential distribution.

# S.4 COD removals

**Fig. S2** COD removals during each feed for MFC_Acetate_ and MFC_Starch._

# S.5 Principle coordinates analysis (PCoA)

**Fig. S3** PCoA plots based on plots based on Bray-Curtis dissimilarity metrics, illustrating microbial community of **a** MFC_Starch_ and **b** MFC_Acetate_ at the end of Feed 12, grouped by electrode row position in MFC (Rows 1-5), with the inoculated electrodes being on Row 1.
